# Case Report: Duodenal neoplasm-induced panniculitis

**DOI:** 10.3389/fmed.2026.1777523

**Published:** 2026-05-18

**Authors:** Hanxiao Mao, Ziyuan Zeng, Lvsha Xie, Yuanmin He

**Affiliations:** Skin Structure and Function Key Laboratory of Luzhou, Department of Dermatology, The Affiliated Hospital of Southwest Medical University, Luzhou, Sichuan, China

**Keywords:** duodenal neoplasms, erythematous nodules, fat necrosis, panniculitis, paraneoplastic panniculitis

## Abstract

Panniculitis comprises a group of diseases characterized by inflammation of the subcutaneous fat, and may present as a distinctive cutaneous paraneoplastic syndrome. Herein, we describe a case of paraneoplastic panniculitis caused by a duodenal neoplasm. The 79-year-old man presented with painful erythematous subcutaneous nodules on the lower extremities without any gastrointestinal symptoms. Histopathological manifestations of the skin included fat necrosis, calcification, and ghost cells. Laboratory test results revealed significantly elevated serum lipase levels (4144.7 U/L). Imaging revealed a normal pancreas, but identified a duodenal tumor with liver metastases. The patient was diagnosed with panniculitis secondary to a duodenal neoplasm with hepatic metastases and died 3 months after diagnosis. This case highlights panniculitis as a warning sign of occult duodenal neoplasms, facilitating clinical diagnosis and intervention.

## 1 Introduction

Panniculitis refers to an inflammatory condition of the subcutaneous fat, and typically presents as tender erythematous nodules. It has a broad differential diagnosis, including infections, autoimmune disorders, and granulomatous disorders (1). Malignancy is a well-documented but rare etiological factor, with most reported cases associated with pancreatic neoplasms (2). Duodenal tumors are uncommon neoplasms that typically present with gastrointestinal symptoms, whereas their clinical manifestation has rarely been reported in association with panniculitis. Herein, we report the case of a 79-year-old man with painful erythematous subcutaneous nodules on the lower extremities, highlight the diagnostic challenges associated with such lesions, and provide the differential diagnosis.

## 2 Case presentation

A 79-year-old man presented with a 1-month history of painful erythematous nodules on the lower extremities. The lesions appeared on the legs without an obvious cause, and progressively increased in number, with the subsequent appearance of pain; therefore, the patient presented to the Department of Dermatology at our hospital. The patient denied fever, cough, abdominal pain, vomiting, melena, or weight loss, and had not received any prior treatment. He was in good health with no history of smoking, alcohol consumption, or drug abuse.

**FIGURE 1:**
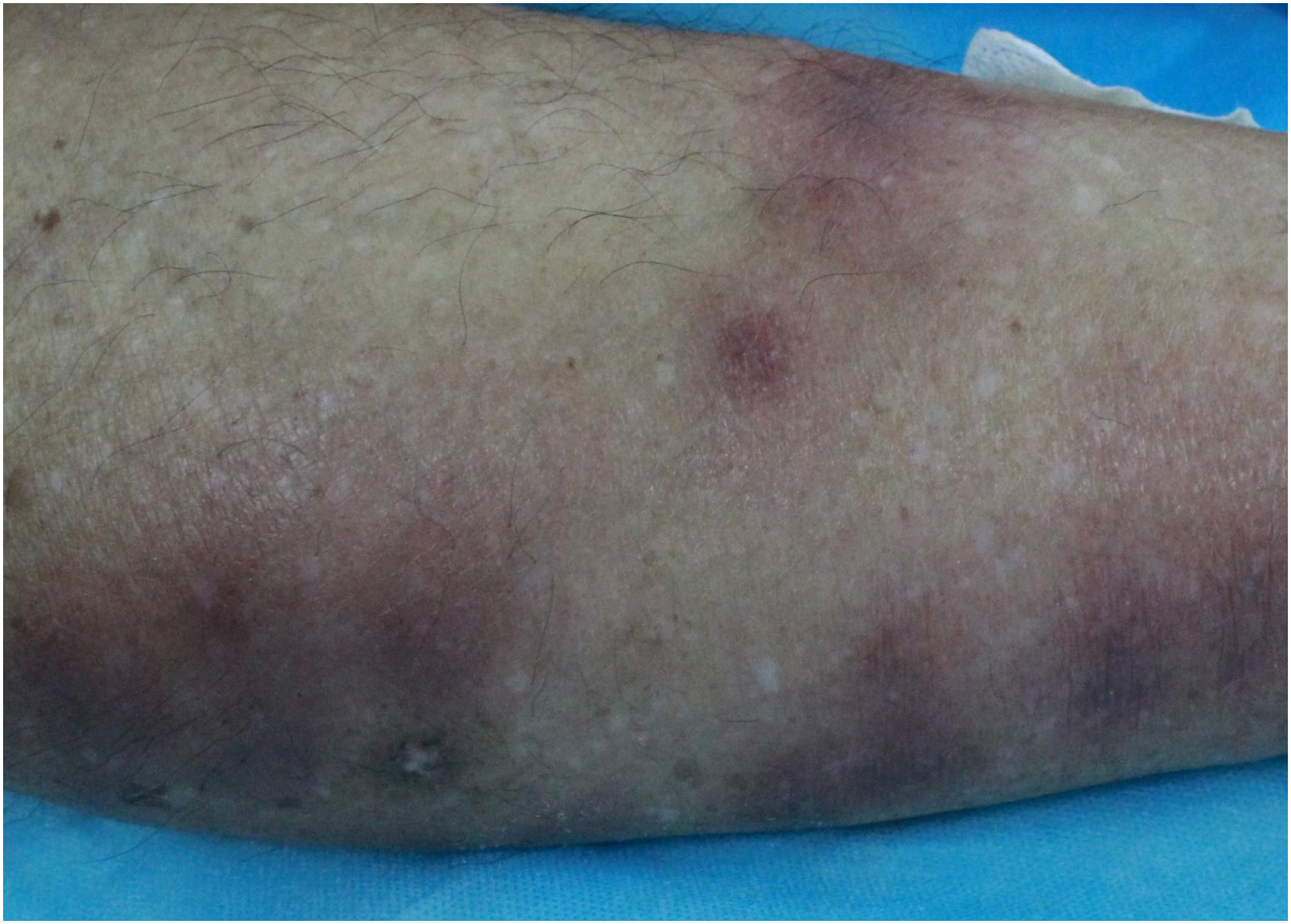
Multiple erythematous subcutaneous nodules on the leg.

On admission, his temperature was 36.6 °C, heart rate was 109 per min, respiratory rate was 20 breaths per min, BP was 146/84 mmHg. Dermatological examination revealed scattered edematous and erythematous subcutaneous nodules on both lower extremities, without visible ulceration or exudation (Figure 1). The lesions were tender upon palpation. Systemic examinations revealed no abnormalities.

**FIGURE 2:**
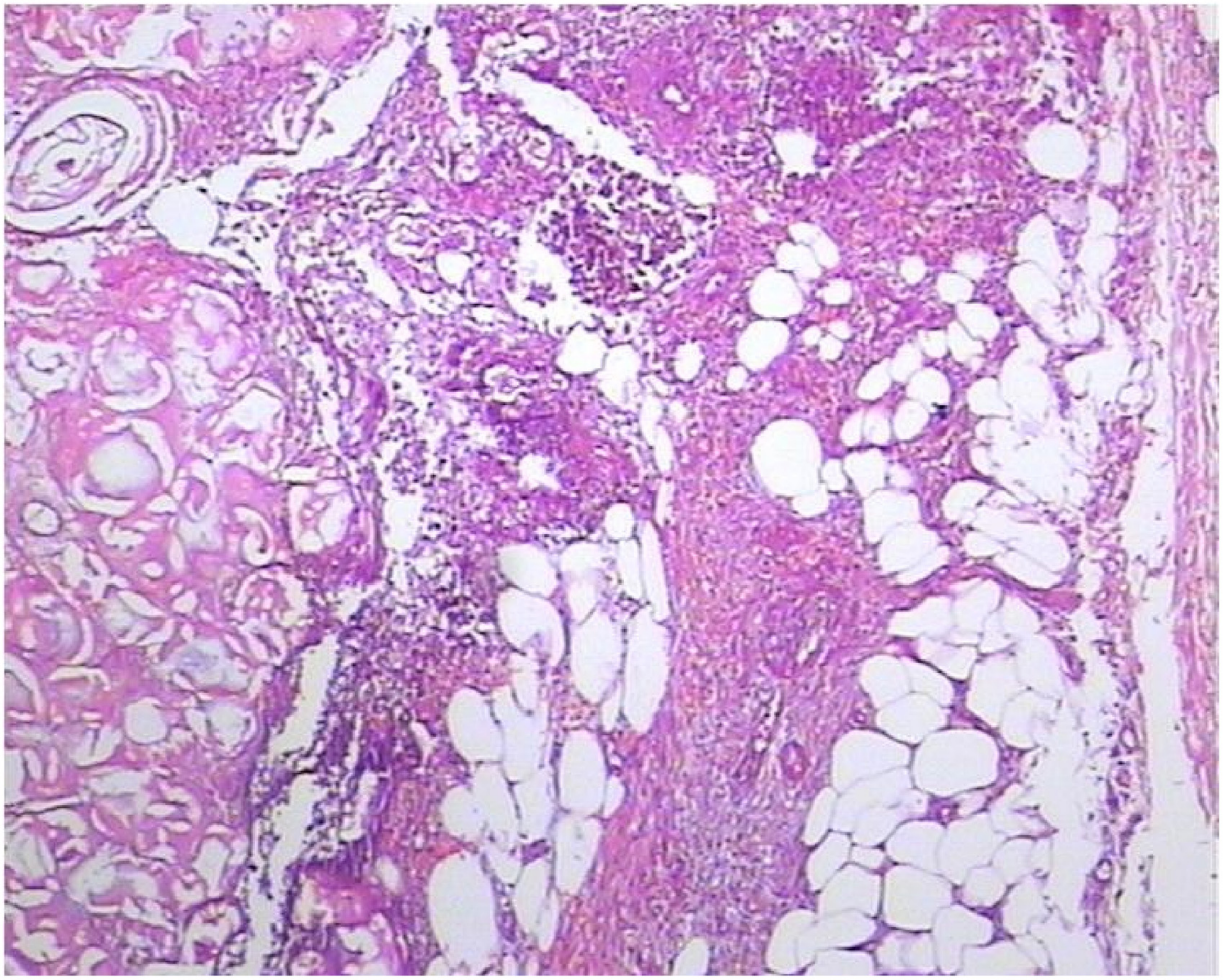
Biopsy from a skin lesion showing predominant lobular panniculitis with focal fat necrosis, “ghost cells”, calcium deposits and surrounding lymphocytic infiltration (HE, ×40).

Laboratory data showed significantly elevated serum lipase levels (4144.7 U/L). No abnormalities were found in complete blood count, procalcitonin levels, hepatic and renal function, electrolytes, coagulation function, or pancreatic amylase levels. Serum gastrointestinal tumor markers, including CEA, AFP, CA199, CA50, CA242, CA724, and PIVKA-II, were negative. Cutaneous histopathology revealed predominantly lobular panniculitis with liquefactive fat necrosis, ghost cells, calcium deposits and surrounding lymphocytic infiltration, findings that are suggestive of pancreatic panniculitis (Figure 2).

**FIGURE 3:**
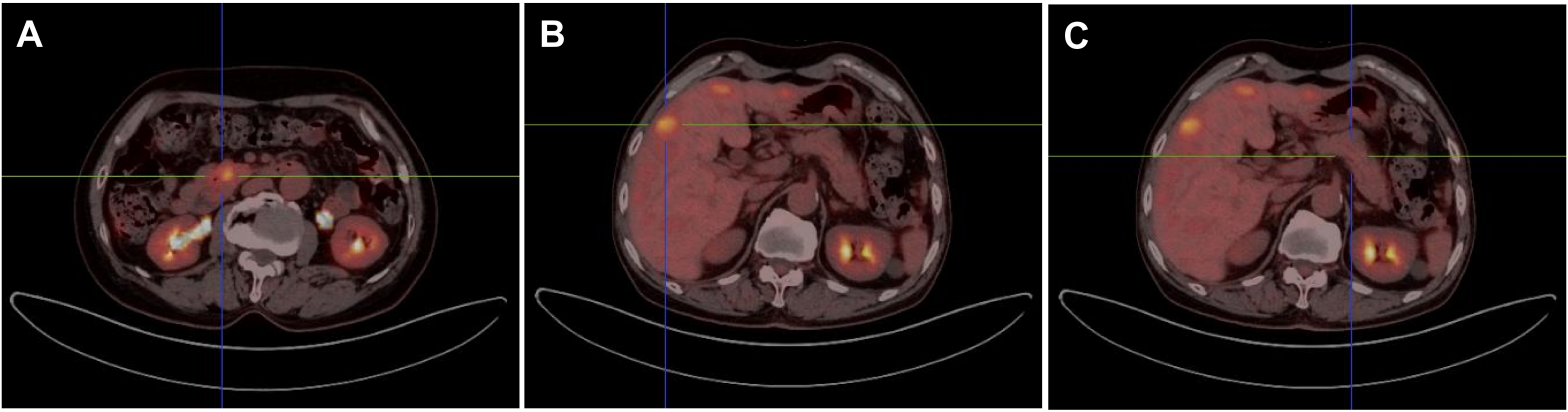
Imaging of the duodenum **(A)**, liver **(B)** and pancreas **(C)** with PET/CT.

To evaluate the systemic disease status, the patient underwent positron emission tomography/computed tomography (PET/CT). The PET/CT findings revealed heterogeneous wall thickening at the duodenal junction between the descending and horizontal segments, accompanied by hypermetabolism, which was considered as a malignant tumor focus (Figure 3A). Additionally, multiple nodular and mass-like lesions within the liver were considered to be caused by tumor metastasis (Figure 3B). Imaging results showed a normal pancreas with no evidence of focal pancreatic lesions (Figure 3C).

Based on the PET/CT, skin biopsy, and laboratory examination results, the patient was ultimately diagnosed with panniculitis due to a duodenal neoplasm with hepatic metastases. The patient refused to undergo recommended diagnostic examinations and therapeutic interventions for personal reasons. Unfortunately, the patient died 3 months after diagnosis.

## 3 Discussion

Panniculitis is a condition characterized by inflammatory nodules or plaques in subcutaneous fat, representing a heterogeneous spectrum of inflammatory processes. Clinically, panniculitis poses considerable diagnostic challenges as it is associated with various diseases. It is most frequently associated with autoimmune, infectious, or traumatic triggers, but reports of its association with malignancy are rare (3–5). Herein, we reported a case of panniculitis secondary to a duodenal neoplasm in a 79-year-old man. This case highlights the importance of considering underlying malignancy in the differential diagnosis of unexplained panniculitis, particularly when it presents with atypical features or is refractory to conventional therapies.

Although established, the association between malignancy and panniculitis is uncommon. Pancreatic carcinoma is the most frequently reported entity causing the classic syndrome of pancreatic panniculitis (6). Other solid neoplasms, including liver carcinoma (7–9), gastric adenocarcinoma (10), and adrenal neuroendocrine carcinoma (11), have been sporadically documented.

The pathogenesis of malignancy-associated panniculitis has not yet been fully elucidated. The classic mechanism often observed in pancreatic primaries is the systemic release of lipolytic enzymes (e.g., lipase and trypsin) by tumor cells, resulting in distant lipolysis and adipocyte necrosis (12, 13). In cases of non-pancreatic tumors such as duodenal carcinoma, tumor-related mechanical compression, direct invasion or metastatic spread to peripancreatic tissues, and lipase production by the tumor itself may induce similar enzymatic release. Alternatively, paraneoplastic mechanisms involving lipase production by tumors and/or tumor-derived cytokines may also play a role (14–17).

In our case, the patient exhibited no gastrointestinal symptoms such as abdominal pain or distension. The subcutaneous nodules in the lower limbs were pathologically consistent with panniculitis. Despite elevated serum lipase levels, imaging revealed no structural abnormalities in the pancreas except for duodenal carcinoma. This combination of elevated lipase and absence of pancreatic structural abnormalities on imaging helped distinguish the presentation from classic pancreatic panniculitis, which typically arises from underlying pancreatic disorders. Furthermore, other causes of lobular panniculitis were excluded based on clinical, histopathological, and laboratory findings, including erythema nodosum, lupus panniculitis, and subcutaneous panniculitis-like T-cell lymphoma. Given this discrepancy and the exclusion of other causes, the subcutaneous nodules in this patient were considered a paraneoplastic presentation of duodenal carcinoma.

Our patient died of cancer progression within 3 months post-diagnosis without receiving anticancer therapy, underscoring the poor prognosis associated with untreated advanced duodenal malignancies. This case suggests that panniculitis can serve as a warning sign of occult duodenal neoplasms, emphasizing its potential diagnostic utility in facilitating timely intervention.

## Data Availability

The original contributions presented in this study are included in this article/supplementary material, further inquiries can be directed to the corresponding author.
